# Commentary: “There’s been a Flaw in Our Thinking”

**DOI:** 10.3389/fimmu.2015.00351

**Published:** 2015-07-16

**Authors:** E. Sally Ward, Raimund J. Ober

**Affiliations:** ^1^Department of Molecular and Cellular Medicine, Texas A&M Health Science Center, College Station, TX, USA; ^2^Department of Microbial Pathogenesis and Immunology, Texas A&M Health Science Center, Bryan, TX, USA; ^3^Department of Immunology, University of Texas Southwestern Medical Center, Dallas, TX, USA; ^4^Department of Biomedical Engineering, Texas A&M University, College Station, TX, USA

**Keywords:** FcRn, IgG, subcellular trafficking, pinocytosis, transport

We thank the editors for the opportunity to address the overstatements in the recent opinion article (1). Over the past two decades, the role of FcRn in regulating the levels and transport of IgG in the body has been established (2–5), validating the insightful prediction of Brambell in the 1960s that IgG is salvaged from catabolism by receptors located within cellular compartments and/or on the surface of cells (6, 7). Remarkably, this hypothesis was made in the absence of knowledge of the molecular details of IgG–FcRn interactions. It is now well known that FcRn binds to IgG at acidic pH (~6.0) with very low or negligible affinity at pH 7–7.4 (8–11), providing an elegant biological solution to achieve exocytic release of recycled IgG. Further, the negligible binding of IgG to FcRn at pH 7–7.4 supports the concept that fluid-phase, pinocytic uptake is the primary mediator of ligand entry into cells exposed to this pH range. However, in the absence of information concerning the pH dependence of FcRn–IgG interactions around 50 years ago, it was not possible to postulate the mechanism of IgG uptake into cells bathed at acidic pH. Notably, in the light of the pH dependence of complex formation, receptor-mediated internalization of IgG for cells at acidic pH is expected to represent a major pathway, although this does not preclude the occurrence of concomitant fluid-phase processes. Consequently, the relative contributions of fluid-phase vs. receptor-mediated pathways for IgG internalization are highly dependent on the pH of the extracellular environment. Further, FcRn biology has been enriched over the past decade by the recognition of its much broader expression pattern and the elucidation of its role in multiple diverse processes, including antigen presentation and mucosal immunity (12–15). Collectively, these developments have motivated multiple *in vitro* cellular studies under conditions designed to emulate the physiological environment of interest.

Numerous analyses of FcRn/IgG trafficking have been performed using cells bathed in medium containing relatively high concentrations (~1–17 μM) of wild type IgG at pH 7.0–7.4 to enable fluid-phase, pinocytic uptake (16–22). Importantly, IgGs that bind with negligible affinity to FcRn accumulate in cells under these conditions (18, 21). Reciprocally, the use of low concentrations (~130 nM) of IgGs that bind to FcRn with the typical pH dependence results in almost background levels of internalization (23). The endosomal sorting of fluorescently labeled wild type IgG in FcRn-expressing endothelial cells has been analyzed at near neutral pH using IgG concentrations (~3–7 μM) that favor fluid-phase uptake (18). These studies demonstrated that IgG is quantitatively routed within sorting (or early) endosomes in association with FcRn into tubulovesicular transport carriers, supporting the concept that sorting endosomes are major sites of FcRn-mediated recycling of IgG following pinocytosis. By contrast, an engineered IgG (H435A mutant) that does not bind to FcRn accumulates in the vacuole of the sorting endosomes and is subsequently delivered to lysosomes. In a related study, exocytic processes involving FcRn and wild type IgG have been characterized at the single molecule level following exposure of cells to relatively high IgG concentrations at pH 7.4 (19). Further, IgG recycling and saturation of FcRn recycling pathways (21, 23, 24) were quantitated under similar conditions. Analyses of the transport of wild type IgG within endothelial, trophoblast and renal epithelial cells have also been performed analogously (16, 17, 20). In light of these studies, the statement advanced by the author of the recent opinion article that “it proved virtually impossible to perform *in vitro* studies of IgG uptake by cultured cells unless the medium was acidic” is perplexing.

In any studies of receptor/ligand trafficking, it is essential to distinguish the behavior of ligand from that of receptor. Considering the negligible affinity of most naturally occurring IgGs for FcRn at near neutral pH, these ligands are unsuitable for use in labeled form as FcRn tracers under these conditions. Consequently, engineered IgG ligands with increased affinity for FcRn at pH ~7 have been used at low concentrations (10–30 nM) that result in negligible fluid-phase pinocytosis (23) to track receptor during endocytosis and trafficking to sorting endosomes (25, 26). Parenthetically, these engineered antibodies compete very effectively with wild type IgG for FcRn binding and therefore have utility as IgG depleting agents in therapy and diagnosis (27, 28). The potential applications of antibodies of this class (“Abdegs”) have motivated analyses of their subcellular trafficking behavior using conditions where receptor-mediated uptake predominates (23, 29).

By contrast with analyses at near neutral pH, experiments have been conducted using acidic pH to mimick the *in vivo* environment corresponding to biological systems for which this is appropriate, such as the apical surface of gut epithelium. These cells are exposed to an acidic microenvironment due to the activity of Na+/H+ exchangers (30). These conditions enable receptor-mediated endocytosis of IgGs at low concentrations that limit fluid-phase accumulation (23, 31, 32). This experimental design results in FcRn-mediated transcytosis and/or recycling [e.g., Ref. (3, 5, 33–35)], and multiple studies including electron tomographic analyses validate the physiological relevance of this approach [e.g., Ref. (31, 36)]. Anderson questions the validity of bathing cells at acidic pH, substantiating his argument with “Gut pH had been measured only once, with litmus paper, and the observation was never repeated.” This statement is surprising, as publications can readily be found in which different techniques demonstrate that the pH of the proximal portion of the intestinal lumen is mildly acidic [pH 6–7 (37, 38)]. For instance, this is well illustrated clinically with the post-pyloric feeding tube placement pH testing in neonates and children (39, 40).

Further, the argument of the author of the recent opinion article that there is a minimal receptor-mediated internalization by (epithelial) cells at acidic pH due to the low proportion of FcRn present on the cell surface relative to intracellular levels neglects consideration of receptor dynamics. Specifically, the low steady state levels of FcRn on the plasma membrane do not exclude the possibility of rapid receptor endocytosis following exocytic events. Indeed, the observation that engineered antibodies with high affinity for FcRn at near neutral pH efficiently accumulate to relatively high levels within cells of multiple different lineages, but only if the cells express FcRn, is consistent with such dynamic cycling behavior (32).

In summary, the primary conclusion that the subcellular pathways taken by IgG following fluid-phase, pinocytic uptake into cells have been ignored for two decades is unfortunately premised on a highly selective review of the literature. To the contrary, a cursory survey of the relevant publications clearly demonstrates that Brambell’s model for regulating IgG homeostasis and transport by receptor-mediated salvage has formed the conceptual foundation to investigate these processes using modern experimental tools. Beyond Brambell’s predictions, the discovery of new and unexpected roles for FcRn has also prompted experiments tailored to specifically investigate the biological questions at hand.

## Conflict of Interest Statement

The authors declare that the research was conducted in the absence of any commercial or financial relationships that could be construed as a potential conflict of interest.
